# Porous Starch Materials via Supercritical- and Freeze-Drying

**DOI:** 10.3390/gels5010012

**Published:** 2019-02-26

**Authors:** Victor Baudron, Pavel Gurikov, Irina Smirnova, Steve Whitehouse

**Affiliations:** 1 Institute of Thermal Separation Processes, Hamburg University of Technology (TUHH), 22073 Hamburg, Germany; pavel.gurikov@tuhh.de (P.G.); irina.smirnova@tu-harburg.de (I.S.); 2 Nestlé Product Technology Centre York, Nestec York LTD, PO BOX 204, York YO91 1XY, UK; Steve.Whitehouse@rdyo.nestle.com

**Keywords:** freeze-drying, supercritical drying, aerogel, starch, open cell foam, cryogel, cryogelation

## Abstract

The production of porous materials based on starch has been explored with supercritical drying—yielding aerogel—and freeze-drying. The two drying procedures were applied on the same gelling solution of amylomaize starch pasted at 140 °C and for two concentrations (5 and 10 wt.%). After gelation and retrogradation, water from the samples to be supercritically dried was exchanged to ethanol. The resulting starch aerogel presented high specific surface area (197 m^2^/g). Freeze-drying was assessed by investigating the effect of the gelation, retrogradation, freezing temperature, and sublimation pressure. The resulting starch materials were macroporous, with limited specific surface area and limited mechanical integrity. Cohesive open cell foam with pore size of ~20 µm was produced by quenching the hot starch melt in liquid nitrogen. The highest specific surface area obtained with freeze-drying was 7.7 m^2^/g for the hot starch melt frozen at −20 °C.

## 1. Introduction

Porous materials based on biopolymers present a tremendous variety of applications such as scaffolds for cell growth, drug carriers [1,2], thermal or acoustic insulation, [3,4] and oil spill remediation [5,6]. The properties required for each application (surface chemistry, surface area, pore volume, and pore size) depend on the starting material as well as on the production process.

Producing a gel and drying it while maintaining their inherent structure and porosity is one path for the production of such materials. Drying a gel with conventional convection drying results in large capillary stresses in the pores at the vapor–liquid interface. This can provoke the collapse of their structure and yield a dense nonporous material, especially with soft biopolymer-based gels.

With the appropriate pore-filling solvent (e.g., ethanol, methanol, and acetone), supercritical drying can be used to avoid the formation of the vapor–liquid interface by operating the drying in the supercritical conditions. Carbone dioxide is often used for the supercritical drying because of its capability to form supercritical mixtures with a wide variety of solvents as well as its mild supercritical conditions, low toxicity and nonflammability. With such an approach, it is possible to produce aerogels that have mesoporous open pore network with high surface area and high porosity.

With freeze-drying, the gels are first frozen, transforming all the pore filling liquid to a solid. The sublimation of the solvent is then achieved at low pressure, avoiding the formation of the vapor–liquid interface. A variety of mesoporous open pore structures were successfully created by freeze-drying with inorganic or organic systems such as silica (up to 760 m^2^/g [7]) or resorcinol-formaldehyde (up to 495 m^2^/g [8]). It has also been proven possible to achieve mesoporous materials with high surface area using freeze-dying of biopolymers such as cellulose [5,9,10,11,12,13] and chitin [14]. There are also numerous porous materials referred to as aerogel, that only present macropores, such as some cellulose [4], pectin/clay hybrids [15], alginate/clay hybrids [16], and starch [17].

Even though the IUPAC definition of aerogel refers to material that are strictly microporous (*d*_pore_ < 2 nm), the use of the term evolved and now also encompasses mesoporous materials (2–50 nm) and is even used for macroporous ones (that would better be described as open cell foams [18], see also discussion by Gurikov and Smirnova [19]). Such open cell foams are often referred to as cryogels, which can be confused with the use of the same term to describe hydrogels produced via cryogelation. Cryogelation, also referred as cryotopic gelation or cryostructuration, is the formation of a gel by a freeze/thaw process [20]. During the freezing of a gelling solution, ice crystals grow and are separated by a nonfrozen liquid where the polymer or other gelling species are concentrated and allowed to gel, cross-link, or react. The ice crystalls continue to grow until touching each others, building a solid frame at the origin of the open pore network in the thawed cryogel [21]. This method is often used for the formation of macropores in hydrogel to support cell growth [22,23] or as a tool in bioseparation [21]. 

Recently, freeze-drying begun to attract interest in the field of aerogel production and there are some direct comparisons between freeze-drying (FD) and supercritical drying (SCD): Deniz et al. compared the SCD and FD for cellulose material based on lupin hull (SCD: 115 m^2^/g vs. FD: 20 m^2^/g) [24]; Buchtová and Budtova and Ganesan et al. for microcrystalline cellulose (SCD: 312 and 303 m^2^/g vs. FD: 62 and 23 m^2^/g) [12,13]; Job et al. for resorcinol-formaldehyde system (SCD: 674 m^2^/g vs. FD: 495 m^2^/g) [8]; and Kenar et al. [25] for starch–palmitate inclusion complex (SCD: 362 m^2^/g vs. FD: 3 m^2^/g). Other review works compared extensively the material properties obtained with FD and SFC across the available literature [18], with possible variation in the gelation methods or formulation as well as variation in the drying methods. 

Starch aerogels have already been produced in the past: Mehling [26], Marco [27], Ubeyitogullari [28], De Marco [29], and Goimil [30] produced starch aerogels with surface area between 60 and 100 m^2^/g; Garcia-Gonzalez [31] and Druel [3] between 220 and 254 m^2^/g; and Kenar, by forming starch–sodium palmitate complexes, reached up to 362 m^2^/g [25]. Starch open cell foams were produced through freeze-drying by Abhari and Qian with sodium citrate cross-linking [17,32], by Mariko with lignocellulose nanofibril reinforcement [33], and by Kenar with starch sodium palmitate inclusion complex [25].

Mikkonen et al. [34] explored the possible applications of bio-based aerogels, mentioning their potential use as a thermal insulation material, for example as a replacement for polystyrene packing. As demonstrated by Druel et al. [3], the low thermal conductivity of starch aerogel (0.021–0.022 W/m·K) makes it a possible candidate for such applications. Additionally, with their large surface area, starch aerogels were adapted for active compound adsorption and controlled delivery, as demonstrated by Garcia-Gonzalez et al. [31], for benzoic acid and ketoprofen, and by Mehling et al. [26] for ibuprofen and paracetamol. 

In this work, we propose a direct comparison of FD and SCD of starch gel and investigate the different morphologies that originate from these two processes.

## 2. Results and Discussion

**Figure 1:**
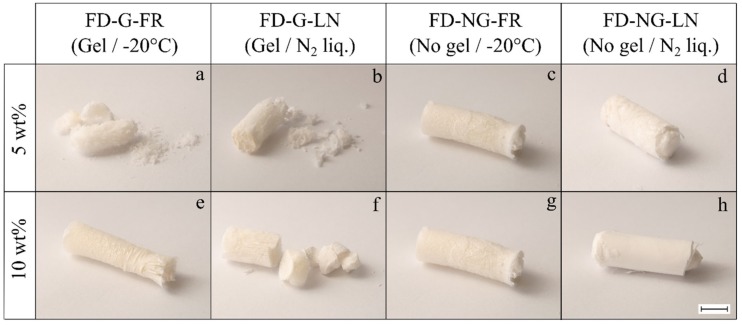
Physical appearance of freeze-dried starch samples (“FD”) derived from 5 and 10 wt.% starch suspensions corresponding respectively to the first and second row of pictures (**a**–**d**) and (**e**–**h**). Samples gelled and retrograded at +6 °C overnight are denoted as “FD-G” (pictures (**a**,**b**,**e**,**f**)), non-gelled samples are denoted as “FD-NG” (pictures (**c**,**d**,**g**,**h**)). Prior to freeze-drying the samples were frozen at −20 °C in a freezer (“FR”—pictures (**a**,**e**,**c**,**g**)) or in liquid nitrogen (“LN”—pictures (**b**,**f**,**d**,**h**)). Sublimation was performed at 0.045 mbar (cold trap temperature −49 °C) for 96 h. The scale bar corresponds to approximatively 1 cm.

**Figure 2:**
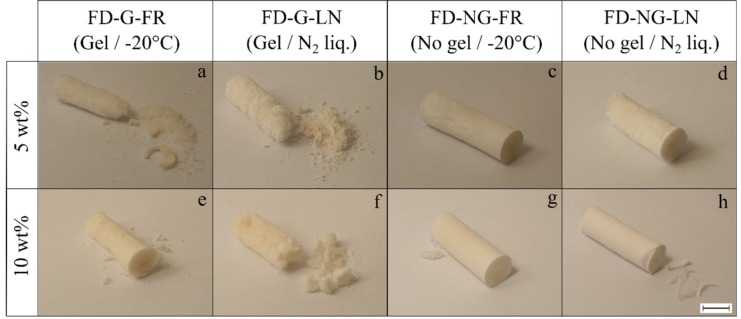
Physical appearance of freeze-dried starch samples (“FD”) derived from 5 and 10 wt.% starch suspensions corresponding respectively to the first and second row of pictures (**a**–**d**) and (**e**–**h**). Samples gelled and retrograded at +6 °C overnight are denoted as “FD-G” (pictures (**a**,**b**,**e**,**f**)), non-gelled samples are denoted as “FD-NG” (pictures (**c**,**d**,**g**,**h**)). Prior to freeze-drying the samples were frozen at −20 °C in a freezer (“FR”—pictures (**a**,**e**,**c**,**g**)) or in liquid nitrogen (“LN”—pictures (**b**,**f**,**d**,**h**)). Sublimation was performed at 2.38 mbar (cold trap temperature −10 °C) for 48 h. The scale bar corresponds to approximatively 1 cm.

The freeze-dried samples that were allowed to form a gel at 6 °C (series “G”) demonstrated friable physical appearance with significant and inhomogeneous shrinkage. These features were evident for both slowly frozen at −20 °C and flash frozen at −196 °C starch gels (series “FR” and “LN”), see Figure 1a,b,e,f and Figure 2. Doubling the starch concentration improved the mechanical integrity only slightly.

The samples prepared by direct freezing of the hot starch melt (series “NG”) yielded monoliths with moderate shrinkage (about 10% of the diameter) and friability, see Figure 1c,d,g,h and Figure 2. The samples of FD-NG-LN that were freeze-dried at 0.045 mbar (Figure 1h and Figure 2h) possessed a smooth and visually homogeneous skin that contrasts with the surface cracks and macropores seen for all other samples.

From the above observations it can be concluded that mechanical integrity is mainly determined by the fact whether or not the pasted starch was gelled prior to freeze-drying.

**Figure 3:**
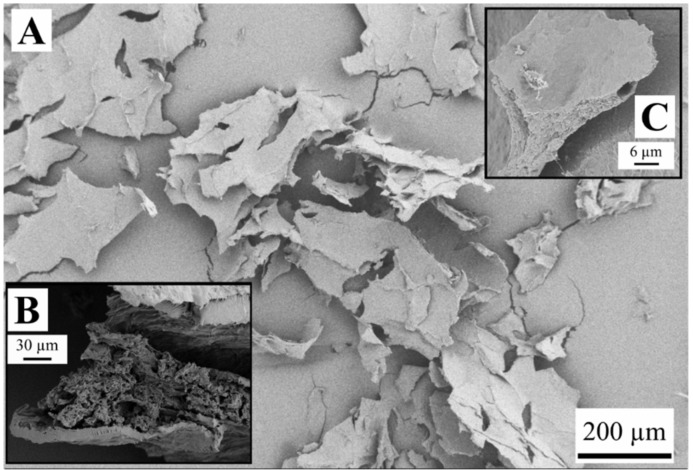
Structure of the sample FD-G-FR derived from 5 wt.% starch suspension and freeze-dried at sublimation pressure of 0.045 mbar (**A**,**C**) and 2.38 mbar (**B**).

**Figure 4:**
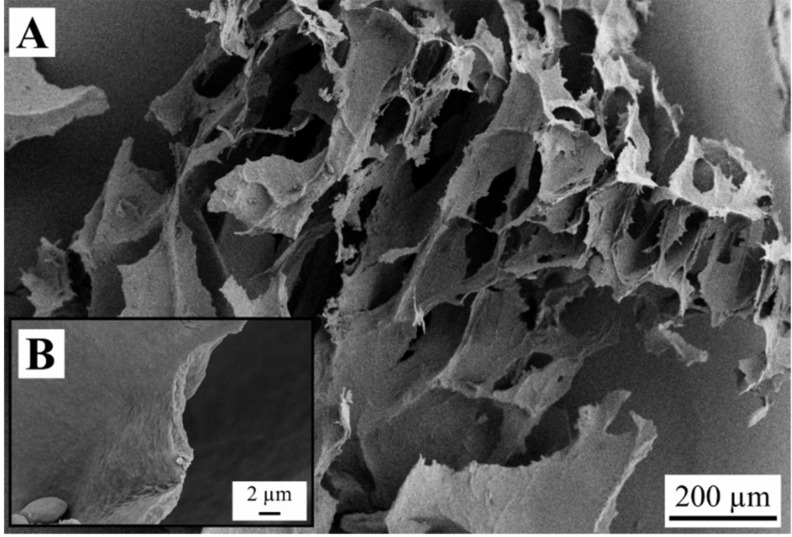
Dense structure of the sample FD-G-LN derived from 5 wt.% starch suspension and freeze-dried at sublimation pressure of 2.38 mbar. Picture (**A**) shows an overview of the material structure and picture (**B**) presents a dense pore wall with higher magnification.

**Figure 5:**
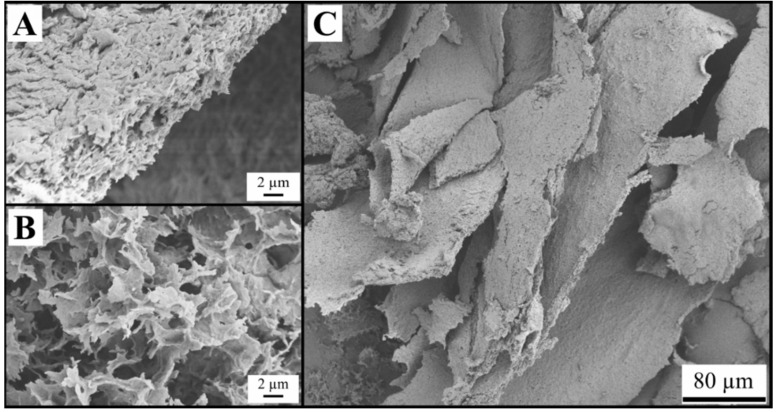
Partially porous structure of the sample FD-G-LN derived from 5 wt.% starch suspension and freeze-dried at sublimation pressure of 0.045 mbar. Picture (**C**) shows an overview of the material structure. Pictures (**A**,**B**) correspond respectively to a pore wall with apparent porosity and a region with smaller pores observed with higher magnifications.

**Figure 6:**
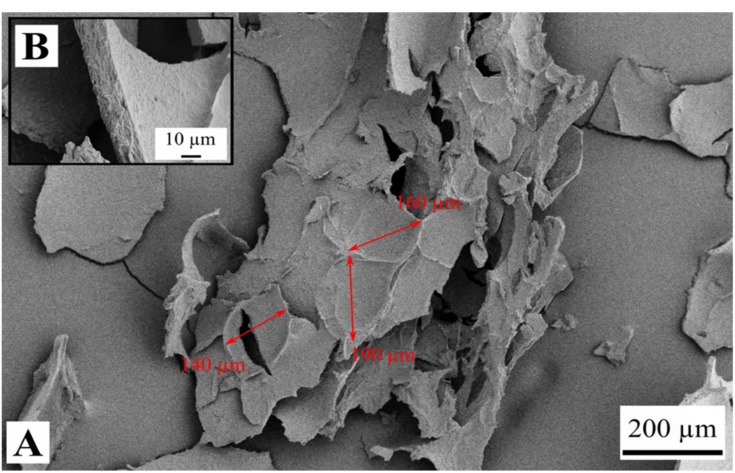
Structure of the sample FD-NG-FR derived from 5 wt.% starch suspension and freeze-dried at sublimation pressure of 2.38 mbar. Picture (**A**) shows an overview of the material structure and picture (**B**) a dense pore wall with higher magnification.

Observations with SEM revealed structures of weakly bound sheets for the samples FD-G-FR and FD-G-LN (Figure 3, Figure 4, Figure 5 and Figure 6). Similar structures were observed for freeze-dried alginate/clay [16], resorcinol-formaldehyde [8], and starch reinforced with lignocellulose fibrils [33].

The sheet-like structures are usually attributed to the slow growth of water crystals that compress the gel network into almost nonporous planar aggregates. Indeed, most of the sheets are dense (Figure 3C and Figure 4B). Only a few remnants of the gel structure can be observed (Figure 3B and Figure 5A,B).

Rapid freezing of the starch gels in liquid nitrogen (samples FD-G-LN, both 5 and 10 wt.%) did not result in a finer structure: mostly dense sheets can be observed in Figure 4A,C. However, in this case some regions were found to be much more porous than others (cf. Figure 5A,B). This inhomogeneity probably arises from the temperature gradient set up in the radial direction when the gel was immersed in liquid nitrogen, with fine ice crystals in the peripheral zone and coarse crystals in the central region. It was natural to expect that slow freezing at −20 °C (sample FD-G-FR) should result in essentially the same morphology with dense walls what was indeed observed in our experiments. It is important to note that both 5 and 10 wt.% suspensions demonstrated similar morphology (data not shown).

**Figure 7:**
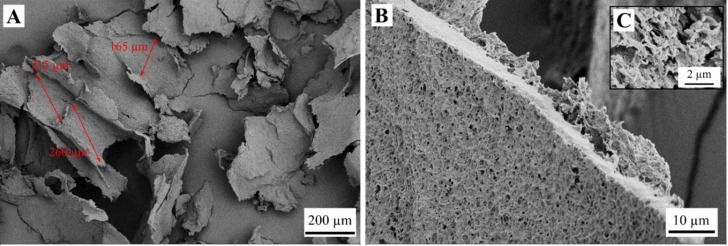
Structure of the sample FD-NG-LN derived from 5 wt.% starch suspension and freeze-dried at sublimation pressure of 0.045 mbar. Picture (**A**) shows an overview of the material structure and pictures (**B**,**C**) a pore wall with higher magnifications

**Figure 8:**
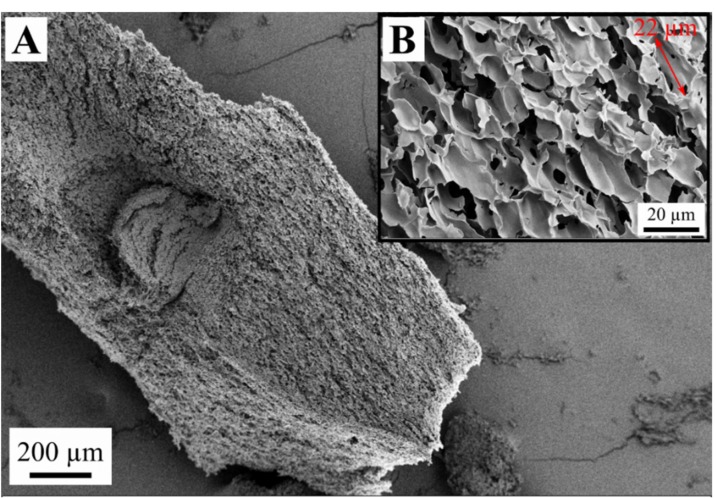
Structure of the sample FD-NG-LN derived from 10 wt.% starch suspension and freeze-dried at sublimation pressure of 0.045 mbar. Picture (**A**) shows an overview of the material which fine structure is displayed with a higher magnification on picture (**B**).

Similarly, when pasted starch (hot melt) was frozen at −20 °C without preliminary gelation and retrogradation (sample FD-NG-FR), ordinary sheet-like structures was generated (Figure 6A) with dense sheets and craters on them with a diameter larger than 100 µm (Figure 6B). In contrast to the above observations, for quenching the pasted starch in liquid nitrogen (sample FD-NG-LN) two distinctly different structures were observed depending on the starch concentration. At low starch concentration, rough (Figure 7A) and relatively dense sheets were obtained (Figure 7B,C), whereas an open cell foam with thin nonporous and interconnected sheets with a typical pore diameter of ~20 µm, was observed with higher starch concentration (Figure 8). Similar structures were reported for freeze-dried nanofibrillated cellulose (frozen at −80 °C), cryogelated gelatin–fibrinogen [23], and potato starch cross-linked by citric acid [17,32].

**Figure 9:**
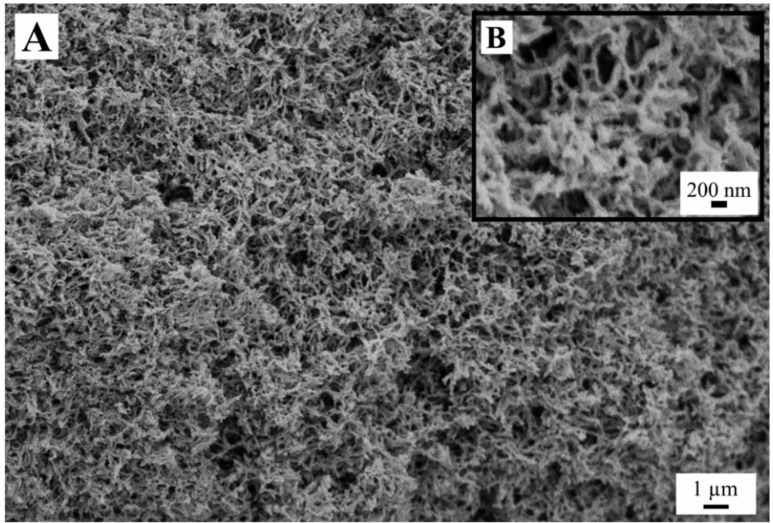
Structure of the starch aerogel (SCD-G) derived from 10 wt.% starch suspension. Picture (**A**) shows an overview of the material which fine structure is displayed with a higher magnification on picture (**B**).

On the contrary, supercritical drying of starch gels derived from both 5 and 10 wt.% (samples SCD-G) resulted in a well-developed interconnected structure with the pore size less than 1 µm and fiber width of less than 100 nm (Figure 9). It is generally believed that supercritical drying preserves the structure of the wet gel, i.e., the structure of aerogels resembles structures of the parent hydrogels [12,13]. For calcium alginate this belief was supported by small-angle X-ray scattering (SAXS) data of pristine hydrogels and corresponding aerogels [35,36]. For other polymers no direct structural comparison was made, to the best of our knowledge.

Leloup et al. [37] studied the microstructure of amylose hydrogels by vitrifying the gel’s water via flash freezing of hydrogel thin slices (10 µm) on a copper plate cooled to −270 °C (with liquid helium). After further preparations, the samples could be observed with SEM and presented structures identical to our starch aerogel observed on Figure 9. The fibrils diameter were measured to be 20 ± 10 nm with a mesh size (distance between fibrils) of several hundreds of nanometers which compare well to the aerogel fibrils diameters (~40 nm) and pore sizes observed. The fibrils proved to be composed of amylose structure presenting B-type X-ray diffraction pattern characteristic of amylose double helices arranged obliquely to the fibril axis. These similarities strongly point out that the starch aerogel also have similar amylose chains structure. Garcia-Gonzalez et al. [31] measured the X-ray diffraction pattern of starch aerogels which presented a band at 2Θ = 15–19° which corresponds to a B-type diffraction pattern [38]. In addition to the previous considerations, these observations reinforce further the results of Robitzer et al. [35,36] stating that the supercritical drying preserves the microstructure present in the hydrogel. 

Jeong and Lim [39] detected a B-type X-ray pattern in freeze-dried starch material gelled at room temperature and frozen at −20°C (similar to our FD-G-FR samples). Testing the other FD materials for crystallinity (with X-ray diffraction or DSC) and presence of B-type diffraction pattern could give further insight on the starch molecular structure and the effect of the different freezing procedure and sublimation pressure.

We can conclude from the results above that initially pasted starch and corresponding gels both yielded weakly connected dense sheet-like structures upon freezing and subsequent freeze-drying. Freezing temperature and sublimation pressure do not have a significant influence on the structure when the starch concentration is low (5 wt.%). At a high starch concentration (10 wt.%) a coherent macroporous structure can only be prepared from the pasted but non-gelled starch and only when flash frozen in liquid nitrogen. Starch gels frozen in liquid nitrogen instead give rise to an ordinary weakly connected structure with dense sheets.

We surmise here that the ice growth in concentrated starch melts is more constrained than in starch gels with the same starch content. This constrained growth yields smaller ice crystals and as a result an open cell coherent structure. As we discuss below, a pronounced shrinkage during the solvent exchange suggests that the wet gels must possess pores larger than those observed in the aerogel samples, i.e., larger than 100 nm (Figure 9). Hence, the question to what extent the growing ice crystals enlarge the gel pores remains open. It is however evident that the sheet-like structure is not present in the wet gel and originates during the freezing of both freshly pasted and gelled samples.

Depending on the target application, one or few textural properties of the dried porous materials should be maximized. For instance, porous materials for thermal insulation should possess mesoporosity to maximize the Knudsen effect and thus to the decrease of the conduction of gaseous phase [40]. This should be achieved at possibly low densities for the thermal insulator to be as light as possible. Oppositely, macropores are beneficial when an enhanced mass transfer is desired, for example, in tissue engineering for bone ingrowth and vascularization [41], while the density does not play a significant role. In contrast, high specific surface areas are required when adsorption on pores is aimed. This is only achievable with extended micro- and mesoporosity. Thus, the understanding how the textural properties are correlated with preparation conditions is essential for the rational material design.

We noted above that the solvent exchange of water to an organic solvent is required for the conversion of starch gels into aerogels. This is also true for the vast majority of other biopolymers. As a rule, the solvent exchange leads to a noticeable shrinkage, especially when gels are exposed to pure organic solvent from the very beginning. Conversely, freeze-drying yields almost shrinkage-free materials. It is also known that the shrinkage is material- and solvent-specific [42,43,44].

**Figure 10:**
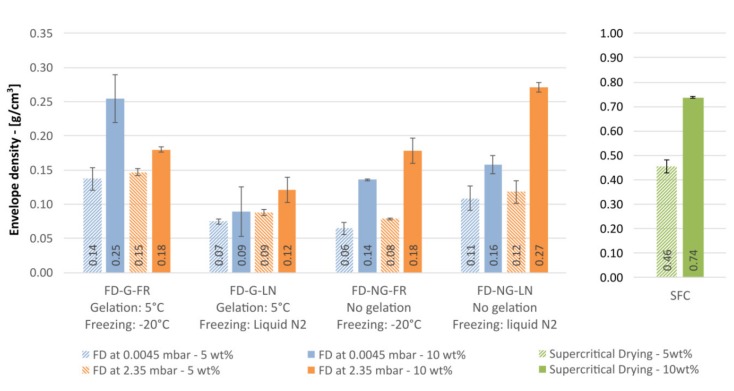
Envelope density comparison of freeze-dried and supercritically dried samples for starch concentration of 5 w% (hashed bars) and 10 wt.% (plain bars).

The simplest measure for the porosity is the envelope density. For the dried starch materials studied here the envelope density is displayed in Figure 10.

First observation is that the measured densities for both freeze- and supercritically dried materials are systematically higher than for hypothetical samples with zero volume shrinkage (“theoretical” densities 0.05 and 0.10 g/cm^3^ assuming no change in solution density). Furthermore, the densities of aerogels are at least by a factor of three larger than that of freeze-dried samples. Qualitatively similar results are reported for cellulose by Buchtová and Budtova [12]. Taking the density as a measure of the shrinkage we can conclude that retrogradation of starch followed by flash freezing in liquid nitrogen results in a minimal shrinkage and porous materials with almost “theoretical” densities. 

Intriguingly, sublimation pressure has a pronounced effect on the resulting density for 10 wt.% starch but not for 5 wt.%. The effect depends on whether starch was retrograded or not. For the retrograded samples frozen at −20 °C (G-FR), sublimation at 0.045 mbar (−49 °C) yielded a ~40% denser material than the sublimation at 2.38 mbar (−10 °C). This effect disappears for the samples frozen in liquid nitrogen (G-LN). The opposite tendency is to be seen for starch frozen right after pasting (samples NG-FR and NG-LN): sublimation at 0.045 mbar always resulted in lighter materials. These findings highlight that the sublimation pressure not only influences the sublimation rate and thus the overall drying time, but also may lead to a reorganization of the porous structure. Whether the effects observed are rather kinetic and related to the drying time (48 vs. 96 h at 2.38 and 0.045 mbar, respectively), or are allied to complex viscosity of the starch gel [45], remains unclear. It is evident that the phenomenon is concentration dependent and warns that a complete preparation protocol should contain the sublimation conditions.

It is to note that starch aerogels demonstrated three times higher densities compared to most dense freeze-dried materials. This observation clearly points to the fact that the preservation of gel structure by supercritical drying does not necessarily mean that the density of resulting aerogels will be lower than for freeze-drying. Due to unavoidable shrinkage, mainly during the solvent exchange, a substantial densification of the structure may occur. Nevertheless, as we discussed above, it is widely believed (and experimentally demonstrated for alginate gels by Robitzer et al. [35,36]) that even the densified structures resemble the structure of the wet gel whereas freezing inherently damages it.

**Figure 11:**
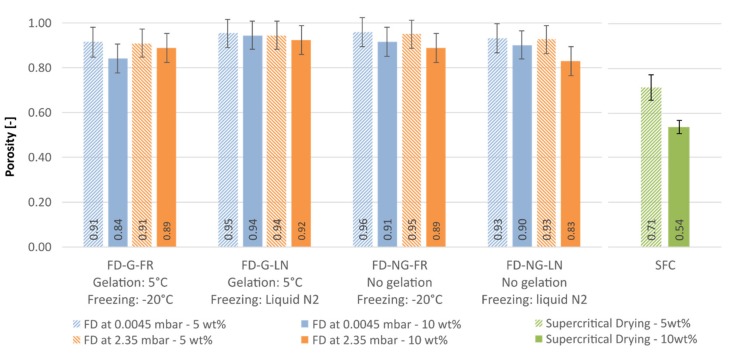
Porosity comparison of freeze-dried and supercritically dried samples for starch concentration of 5 w% (hashed bars) and 10 wt.% (plain bars).

Porosity is the quantity directly related to the geometrical density, see Figure 11. Required skeletal density for all samples was found to be in the range of that for native starch (1.50 g/cm^3^). All freeze-dried samples are falling in the same porosity range with an average porosity of 92 ± 3%, see Figure 11. As expected from the data for the envelope density, the aerogels have lower porosity; 61 and 76% for the 10 and 5 wt.% samples, respectively. Although envelope densities and porosities are directly proportional, the effect of the process conditions is much more visible when the envelope density is reported (cf. Figure 10 and Figure 11). Thus, the envelope density should be preferred when data for highly porous materials reported. 

Nitrogen adsorption was used to measure the specific surface are with the Brunauer–Emmett–Teller (BET) isotherm. Only four freeze-dried samples had a measurable BET surface, which are reported together with the aerogel ones in Table 1. The maximum measured BET surface area (S_BET_) of the freeze-dried sample does not go beyond 7.7 m^2^/g, far behind the starch aerogel that exhibit 183 and 197 m^2^/g. for 5 and 10 wt.%, respectively. 

Both the samples FD-NG-LN and FD-G-FR presented measurable surface areas of 3.4 and 7.7 m^2^/g, respectively. Apparent porosity could also be observed in their walls on some SEM pictures (Figure 3 and Figure 5), hinting that most of the measured surface area might originate from some remaining porosity in the walls. A similar case was illustrated by Job et al. with the resorcinol-formaldehyde system where SEM pictures only show large macropores in the 200 µm range for material that presented high specific surface area up to 495 m^2^/g [8]. 

The sample FD-NG-FR scored the second highest measured BET surface area (6.2 m^2^/g) but no porosity could be observed on the SEM pictures. As stated before and discussed by Jimenéz et al. [4], the final samples have strong anisotropic properties. The absence of wall porosity on the SEM pictures in spite of the measured BET surface could originate from a sampling bias during the SEM sample preparation illustrating that spatially resolved sampling (e.g., border and center) is critical in the assessment of such materials. 

Jimenéz-Saelices et al. demonstrated how the freezing speed was crucial in the freeze-drying of nanofibrillated cellulose; a slow freezing produced material with less than 1 m^2^/g [4] while using a spray based process to achieve a quick freezing yielded large surface area up to 100 m^2^/g [9]. In our case, the effect of the flash freezing in liquid nitrogen was undeniable on the mechanical integrity and on the macropores structure (20 µm open cell foam) of the ungelled samples (FD-NG-LN) but did not extend to the gelled sample (FD-G-LN) illustrating the interdependence of the freezing and gelling processes. Additionally, the highest BET surface obtained with freeze-drying were achieved with the freezing procedure at −20 °C also hinting for the necessity for a balance between the gelation and freezing rate to preserve some mesopore in the walls.

The sublimation pressure, additionally to influence the envelope density as previously discussed, also seems to affect strongly the BET surface area. Indeed, almost no sample freeze-dried at 2.38 mbar produced measurable BET surface area. Additionally, the samples FD-G-FR and FD-NG-FR presented measurable surface area for both sublimation pressures, but the surface area of the samples processed at 2.38 mbar was almost ten times lower for both cases.

Finally, the starch aerogel produced in this study had specific surface area far beyond the ones of the freeze-dried sample and comparable to the ones produced by Garcia-Gonzalez and Smirnova (234 m^2^/g) [31] and Druel et al. (254 m^2^/g) [3], but significantly lower than the starch–sodium palmitate inclusion complex from Kenar (362 m^2^/g) [25].

Starch foams similar to our FD materials were produced via steam explosion by Glenn and Ort [46] and via extrusion of superheated starch melt by Nabar and Narayan [47]. Both studies explored applications of the resulting starch foams as a biodegradable packaging material. Further mechanical testing of our most promising samples should be done to test their compatibility for such applications and reinforcement of the FD starch open cell foam could be considered. Indeed, Chen et al. [15] demonstrated improved mechanical properties of pectin based open cell foams with the addition of clay, and Yildirim et al. [48] improved starch open cell foams strength with the addition of cellulose nanofibrils. Finally, Abhari et al. [17] successfully tested starch open cell foam for the controlled release of hexenal for yeast control in pistachios, opening potential applications in active packaging.

## 3. Conclusions

Even though the mechanical integrity of the freeze-dried material was poor when prepared from the freezing of a gel, direct freezing of the hot starch melt yielded monolithic materials with high porosity and low shrinkage.

Open cell foams with macropores in the 20 µm range were achieved by quenching the 10 wt.% starch melt in liquid nitrogen while the other samples only presented macropores above 100 µm.

Surprisingly, the samples with the higher surface area were obtained by freezing starch gel or paste at −20 °C and did not correspond to the samples with the best mechanical integrity or the smallest macropores. It was demonstrated that macropores are formed during the freezing step and could not be observed for aerogels from supercritical drying.

The sublimation pressure also had a large influence on the envelope densities and surface area, illustrating that the resulting material properties depends on the gelation rate, freezing rate but also on the freeze-drying conditions.

On the other hand, supercritically dried samples displayed specific surface area up to 197 m^2^/g, far beyond what could be achieved with freeze-drying (max 7.7 m^2^/g). Because of significant shrinkage during the solvent exchange step, the aerogel presented lower porosity but had a pore size below 1 µm.

One possible extension of the reported work might include a freeze-thaw process to form macropores in the hydrogel (similar to cryogelation) followed by a solvent exchange and supercritical drying to preserve eventual mesopores in the walls. This could help study the effect of freezing and gelling rate separately from the freeze-drying conditions and could eventually yield materials with two distinct porosity scales. Additionally, the spraying of the hot starch melt into a cold mold [7,9] might allow for smaller macropores.

## 4. Materials and Methods

Amylomaize starch with an amylose content of ~67 wt.% was furnished by Roquette^®^ (Lestrem, France) and used together with deionized water to produce starch suspensions. For the solvent exchange denatured ethanol (99.8 wt.%) was purchased from Carl Roth GmbH (Karlsruhe, Germany).

At the pasting step, five or ten wt.% starch suspensions were produced in cold deionized water under mild stirring. It was then poured into an autoclave equipped with a PTFE container, electrical heating, and magnetic stirring. The temperature was ramped up to 140 ± 5 °C in 30 min, held up for 20 min, and decreased to 95 °C before opening. The starch melt was then poured into cylindrical molds, 1.25 cm in diameter, capped with paraffin film, and further processed for supercritical or freeze-drying. 

For the supercritical drying, the samples were placed directly in a fridge and allowed to gel and retrograde at 6 °C overnight. The starch gel monoliths were unmolded and water was exchanged with ethanol by successive immersions in pure ethanol (gel-to-ethanol ratio approximately 1:5 mL/mL) until its bulk concentration was measured to be above 98 wt.% by a density meter (DMA 4500 M, Anton Paar, Graz, Austria). The samples were then supercritically dried in an autoclave with a volume of 250 mL under continuous flow of CO_2_ (20 g/min) at 120 bar and 60 °C for three hours.

Prior to freeze-drying, the samples were processed according to four different methods to observe the effect of gelation and retrogradation, the freezing temperature, and the pressure during freeze-drying.

Directly after the starch pasting, when no gel is yet formed, some samples were allowed to gel and retrograde at 6 °C overnight before being frozen, while the others were frozen directly with two freezing methods: in a freezer at −20 °C or by immersion in liquid nitrogen (−196 °C). Finally, frozen samples were freeze-dried using Alpha 1-2 LDplus (Christ, Osterode, Germany) at 2.38 mbar (−10 °C in cold trap) for 48 h or at 0.045 mbar (−49 °C in cold trap) for 96 h. Desorption of the remaining water is carried out under vacuum at 60 °C for 24 h (Quantachrome FloVac Degasser; Anton Paar, Graz, Austria). The latter step is also required for the subsequent nitrogen adsorption measurements.

BET surface was measured with nitrogen adsorption (NOVA 4000e, Quantachrome Instrument; Anton Paar, Graz, Austria). Microstructure of the porous starch materials was studied by scanning electron microscopy (Leo Gemini 1530, Zeiss, Oberkochen, Germany) after sputtering with 10 nm gold.

The envelope density ρ_env_ for cylindrical samples was calculated from the sample weight and dimensions (diameter and length). The skeletal density ρ_skel_ was measured with Helium pycnometry (Micromeritics 1305, Aachen, Germany). The porosity was then calculated according to Equation (1).

(1) $$\epsilon =1-\frac{\rho_{\mathrm{env}}}{\rho_{\mathrm{skel}}}$$

Density measurements were conducted in triplicates and reported as the mean ± standard deviation. Uncertainty of the porosity was estimated as follows: One thousand normally distributed pairs were generated for the envelope and skeletal densities with the mean and the standard deviation measured experimentally. The porosity was calculated for each pair resulting in 1000 values. They found to be distributed approximately normally, and the standard deviation of the resulting distribution was used as a measure for uncertainty of the porosity.

The following labels are used: drying method is denoted as “FD” and “SCD” for freeze- and supercritical drying, respectively. The samples that allowed to gel and retrograde at +6° C overnight are labels as “G”, whereas for non-gelled pasted starch the notation “NG” is used. For freeze-dried samples the freezing regime is specifies as “FR” for freezer (−20 °C) and “LN” for liquid nitrogen (−196 °C).

## Figures and Tables

**Table 1:** Brunauer–Emmett–Teller (BET) surface area of the porous starch materials produced with freeze-drying and supercritical drying measured with nitrogen adsorption.

**Sample**	**Starch Concentration [wt.%]**	**S_BET_ [m^2^/g]**
FD-G-FR/2.38 mbar	5	0.6
FD-G-FR/0.045 mbar	5	7.7
FD-NG-FR/2.38 mbar	10	0.8
FD-NG-FR/0.045 mbar	10	6.2
FD-NG-LN/0.045 mbar	10	3.4
SFC Aerogel	5	183
SFC Aerogel	10	197

## Abbreviations

d_pore_	[nm]	Pore diameter
ϵ	[-]	Porosity
ρ_env_	[kg/m^3^]	Envelop density
ρ_skel_	[kg/m^3^]	Skeletal density
S_BET_	[m^2^/g]	Specific surface area measured with the BET isotherm
wt.%	[-]	Weight percent
BET	Brunauer–Emmett–Teller	
FD	Freeze-drying	
FR *	Sample frozen in freezer at −20 °C	
G *	Sample gelled before freezing	
LN *	Sample frozen in liquid Nitrogen	
NG *	Sample frozen without prior gelation	
SEM	Scanning electron microscope	
SFC	Supercritical drying	
* Further details on sample denomination can be found at the end of the Section 4 Material and Methods.		
